# Differential association of plasma monocyte chemoattractant protein-1 with systemic inflammatory and airway remodeling biomarkers in type-2 diabetic patients with and without asthma

**DOI:** 10.1186/s40200-016-0264-4

**Published:** 2016-09-29

**Authors:** Sardar Sindhu, Merin Koshy, Areej Abu Al-Roub, Nadeem Akhter, Saad Al Zanki, Shamsha Ali, Sriraman Devarajan, Rasheed Ahmad

**Affiliations:** 1 Immunology & Innovative Cell Therapy Unit, P.O. Box 1180, Dasman, 15462 Kuwait; 2 Tissue Bank Facility, Dasman Diabetes Institute (DDI), P.O. Box 1180, Dasman, 15462 Kuwait

**Keywords:** Type-2 diabetes, Asthma, Monocyte chemoattractant protein-1, Inflammation, Biomarkers

## Abstract

**Background:**

Chronic inflammation is a hallmark of type-2 diabetes (T2D) and asthma. Monocyte chemoattractant protein (MCP)-1 or CCL-2 is a key regulator of monocytic infiltration into the sites of inflammation. The changes in systemic MCP-1 levels and its relationship with other inflammatory/immune markers in T2D patients with asthma remain unclear and have been addressed in this study.

**Methods:**

Plasma samples from 10 asthmatic T2D patients (Group I: BMI = 37.82 ± 9.75 kg/m^2^), 13 non-asthmatic T2D patients (Group II: BMI = 32.68 ± 4.63 kg/m^2^), 23 asthma patients without T2D (Group III: BMI = 30.14 ± 6.74 kg/m^2^), and 25 non-asthmatic non-diabetic controls (Group IV: BMI = 27.99 ± 5.86 kg/m^2^) were used to measure levels of MCP-1 and multiple cytokine/chemokine biomarkers with bead-based multiplex assays using Luminex technology. IgE/ECP were measured using commercial ELISA kits. Data (mean ± SEM) were compared using unpaired Student’s *t*-test and linear dependence between two variables was assessed by Pearson’s correlation coefficient (r) and *P* ≤ 0.05 was considered as significant.

**Results:**

Plasma MCP-1 levels were significantly higher in Group I (337.95 ± 46.40 pg/mL) as compared with Group II (216.69 ± 17.30 pg/mL), Group III (251.76 ± 19.80 pg/mL), and Group IV (223.52 ± 133.36 pg/mL). MCP-1 showed differential association with tested biomarkers by correlating positively with: (i) IFN-α2, IL-10, fractalkine, and VEGF in T2D patients with asthma; (ii) IL-6 and GRO-α in T2D patients without asthma; (iii) MDC, IP-10, GM-CSF, FGF-2, and PDGF-AA/BB in patients with asthma only; and (iv) FPG and TG in non-asthmatic non-diabetic controls. MCP-1 associated with IL-1RA only in subjects with asthma.

**Conclusion:**

The systemic MCP-1 levels were significantly elevated in T2D patients with asthma as compared with those without asthma and/or diabetes while these changes correlated differentially with important biomarkers of inflammation and airway remodeling.

## Background

The increasing evidence suggests that chronic inflammation is a critical factor involved in type-2 diabetes (T2D) and asthma [1–5]. Monocyte chemoattractant protein (MCP)-1, also known as C-C chemokine ligand (CCL)-2, recruits monocytes to the sites of inflammation, such as into the expanding adipose tissue in obesity/T2D [6, 7] or the arterial vessel wall in asthmatic patients [8]. MCP-1, which is associated with a myriad of diseases including allergic asthma, multiple sclerosis, rheumatoid arthritis, atherosclerosis, and insulin-resistant diabetes, is produced by many cell types such as endothelial and epithelial cells, fibroblasts, smooth muscle, mesangial, and microglial cells, however, predominantly by monocytes/macrophages [9].

The lung is also a target organ for diabetic microangiopathy and the reduced lung function has been frequently observed in diabetic patients due to biochemical changes in the connective tissue as well as non-enzymatic glycosylation of proteins induced by chronic hyperglycemia [10, 11]. Thus, the risk of asthma is elevated in the obese by 2–3 times [12] and in the T2D patients by 2 times [13] as compared with normal subjects. Obesity seems to be the major contributor to the pathogenesis of asthma and the increase in abdominal adiposity negatively impacts the lung volumes and pulmonary and bronchial expansions during inhalation. Notably, obesity and T2D are also characterized by oxidative stress and low-grade chronic inflammation [14]. Since MCP-1 is essentially involved in the pathogenesis of several inflammatory conditions, we hypothesized that it could be a major player of predictive significance and a sensitive biomarker in T2D patients with asthma that are also more likely to develop poor disease prognosis. Therefore, the study objective was to measure the plasma MCP-1 levels and assess their relationship with various inflammatory, airway remodeling, and other immune biomarkers in T2D patients with and without asthma. This study also included non-diabetic asthmatic individuals and non-diabetic non-asthmatic subjects as controls. Herein, we report the significantly higher MCP-1 levels in asthmatic as compared with non-asthmatic T2D pateints. Furthermore, plasma MCP-1 levels associated differentially with inflammatory and lung tissue remodeling biomarkers when compared between asthmatic T2D patients and other study groups.

## Methods

### Study population

This study comprised of 23 diabetic and 48 non-diabetic male/female individuals, further subclassified into 4 groups as follows: Group I including 10 T2D patients with asthma (age 53.60 ± 7.78 years; BMI 37.82 ± 9.75 kg/m^2^); Group II including 13 T2D patients without asthma (age 50.92 ± 6.42 years; BMI 32.68 ± 4.63 kg/m^2^); Group III including 23 asthmatic non-diabetic individuals (age 38.70 ± 9.34 years; BMI 30.14 ± 6.74 kg/m^2^); and Group IV including 25 non-asthmatic non-diabetic individuals as controls (age 35.96 ± 8.27 years; BMI 27.99 ± 5.86 kg/m^2^). Written informed consent was obtained from all participants before enrolment in the study and the study protocol was approved by Ethical Review Committee of Dasman Diabetes Institute, Kuwait. Those of age <18 years. or >65 years. or those suffering from serious cardiovascular, hepatic, or renal disease, hematologic or immune disorder, type-1 diabetes, pregnancy, or malignancy were excluded. Clinical and anthropometric characteristics of study participants are summarized in Table 1.

**Table 1:** Characteristics of study population

Parameter	Diabetic		Non-diabetic	
	Asthmatic	Non-asthmatic	Asthmatic	Non-asthmatic
Total number (N)	10	13	23	25
Age (Yrs.)	53.60 ± 7.78	50.92 ± 6.42	38.70 ± 9.34	35.96 ± 8.27
BMI (kg/m^2^)	37.82 ± 9.75	32.68 ± 4.63	30.14 ± 6.74	27.99 ± 5.86
FPG (mmol/L)	9.01 ± 2.57	8.00 ± 2.57	5.37 ± 0.60	5.09 ± 0.65
HbA1c (%)	8.60 ± 1.25	8.20 ± 1.85	5.50 ± 0.42	5.46 ± 0.49
Total cholesterol (mmol/L)	4.68 ± 0.44	5.03 ± 1.00	4.86 ± 0.86	4.70 ± 0.62
HDL (mmol/L)	1.21 ± 0.32	0.99 ± 0.25	1.20 ± 0.31	1.49 ± 0.73
LDL (mmol/L)	2.82 ± 0.56	3.12 ± 1.13	3.09 ± 0.88	2.93 ± 0.61
TG (mmol/L)	1.44 ± 0.72	1.80 ± 1.21	1.46 ± 1.65	0.82 ± 0.45

*BMI* body mass index, *HbA1c* glycated hemoglobin, *FPG* fasting plasma glucose, *HDL* high-density lipoprotein, *LDL* low-density lipoprotein, *TG* triglycerides

### T2D/asthma diagnosis, anthropometric measurements and biochemical analyses

T2D diagnosis was based on results of (i) fasting plasma glucose (FPG), (ii) oral glucose tolerance test (OGTT), and (iii) glycated hemoglobin (HbA1c) test. FPG levels of ≥126 mg/dL (≥7 mmol/L), 2 h-OGTT values of >200 mg/dL (11.1 mmol/L), and/or HbA1C levels of ≥6.5 % on two separate tests were diagnosed by attending physician as T2D. The diagnosis of asthma was based on a compatible clinical history along with an evidence of reversible airflow limitation i.e., increase in forced expiratory volume in 1 s (FEV_1_) of ≥15 % following a bronchodilator or airway hyper-responsiveness i.e., provocative concentration of methacholine causing a 20 % reduction in FEV_1_. Pulmonary function testing and spirometry were performed according to the standards of the American Thoracic Society [15]. Regarding anthropometric/physical data, height and weight were measured while barefoot, waist circumference was measured and the waist-to-hip ratio was calculated. BMI was calculated as follows: body weight (kg)/height (m^2^). An average of the 3 blood pressure readings (Omron HEM-907XL digital automatic sphygmomanometer, Omron Healthcare Inc. IL, USA), taken after 5–10 min rest for each, was obtained. The whole body composition including body fat percentage, soft lean mass and total body water was assessed (IOI 353 Body Composition Analyzer, Jawon Medical, South Korea). Plasma or serum samples were analyzed for FPG, HbA1c, total cholesterol, high- and low-density lipoproteins (HDL, LDL), and triglycerides (TG) levels using standard methods. Briefly, FPG was measured using hexokinase method, HbA1c was measured using Variant device (BioRad, Hercules, CA, USA), serum cholesterol by cholesterol oxidase-peroxidase-amidopyrine method, HDL was measured by direct method using polyethylene glycol-pretreated enzymes, LDL was measured using Friedewald formula, and serum triglycerides were measured using glycerol phosphate oxidase-peroxidase-amidopyrine method.

### Measurement of plasma analytes

A wide range of plasma analytes classified as cytokines, chemokines, activation and growth factors were measured using Milliplex MAP human cytokine/chemokine magnetic bead panel-immunology (Cat.# HCYTOMAG-60 K, Milliplex Corp, USA) multiplex immunoassay while some analytes were measured using commercial enzyme-linked immunosorbent assays (ELISA) such as immunoglobulin (Ig)-E (Human IgE Platinum ELISA kit, Cat.# BMS2097, eBioscience, Austria), and Eosinophil cationic protein (ECP) (Human ECP kit, Cat.# SK00128-01, Aviscera Bioscience Inc. CA, USA) and following the manufacturers’ instructions. The tested analytes included interferon (IFN)-α2, IFN-γ, interleukin (IL)-1α/β, IL-1 receptor agonist (IL-1RA), IL-2, IL-3, IL-4, IL-5, IL-6, IL-7, IL-8, IL-9, IL-10, IL-12p40/p70, IL-13, IL-15, IL-17A, transforming growth factor (TGF)-α, tumor necrosis factor (TNF)-α/β, macrophage chemoattractant protein (MCP)-1, MCP-3, macrophage inflammatory protein (MIP)-1α or CCL-3, MIP-1β/CCL-4, regulated on activation normal T-cell expressed and secreted (RANTES)/CCL-5, eotaxin/CCL-11, macrophage-derived chemokine (MDC)/CCL-22, growth-regulated oncogene (GRO) or CXC chemokine ligand (CXCL)-1, fractalkine/CX3CL-1, IFN-γ-inducible protein (IP)-10/CXCL-10, epidermal growth factor (EGF), FMS-like tyrosine kinase-3 ligand (FLT-3 L), granulocyte colony-stimulating factor (G-CSF), granulocyte-macrophage colony-stimulating factor (GM-CSF), fibroblast growth factor (FGF)-2, platelet-derived growth factor (PDGF)-AA/BB, vascular endothelial growth factor (VEGF), soluble CD40 ligand (sCD40L), ECP, and IgE. Data were acquired using Luminex xMAP analyzer (Luminex 100/200 Milliplex Analyzer, Luminex Corp. USA) following the manufacturer’s instructions while a digital processor managed data output and Milliplex analyst software was used to determine mean fluorescence intensity and analyte concentrations (pg/mL).

### Statistical analysis

For statistical analysis of the data (mean ± s.e.m.), group means were compared using unpaired Student’s *t*-test and linear dependence between two variables was assessed by Pearson’s correlation coefficient (r) using GraphPad Prism software (version 6.05; San Diego, CA, USA); all *P*-values ≤0.05 were considered statistical significant.

## Results

### MCP-1 levels are significantly higher in diabetic pateints with asthma

**Fig. 1:**
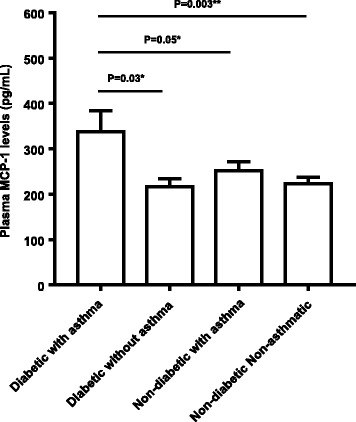
Increased plasma monocyte chemoattractant protein (MCP)-1 concentrations in type-2 diabetes (T2D) patients with asthma. Plasma samples from 10 T2D patients with asthma, 13 T2D patients without asthma, 23 asthma patients with no diabetes, and 25 non-diabetic non-asthmatic controls were used to measure MCP-1 concentrations (pg/mL) using Milliplex MAP human cytokine/chemokine magnetic bead panel multiplex immunoassay as described in Methods. The data, represented as group means with SEM, show that plasma MCP-1 concentrations in T2D patients with asthma (337.95 ± 46.40 pg/mL) were significantly higher than those of T2D patients without asthma (216.69 ± 17.30 pg/mL *P* = 0.03), asthma only individuals (251.76 ± 19.80 pg/mL *P* = 0.05), and non-diabetic non-asthmatic controls (223.52 ± 13.36 pg/mL *P* = 0.003)

T2D is a metabolic disorder involving chronic low-grade systemic inflammation while asthma is a chronic allergic inflammatory disease of the lung. We asked whether the plasma levels of MCP-1/CCL-2, which is a signature inflammatory marker, were elevated in T2D patients with asthma as compared with those having either diabetes or asthma or none of the morbid condition. To this end, as shown in Fig 1, plasma MCP-1 levels were found to be significantly higher in T2D patients with asthma (Group I: 337.95 ± 46.40 pg/mL) as compared with other groups, such as T2D patients without asthma (Group II: 216.69 ± 17.30 pg/mL; *P* = 0.03), non-diabetic individuals with asthma (Group III: 251.76 ± 19.80 pg/mL; *P* = 0.05), and non-diabetic non-asthmatic controls (Group IV: 223.52 ± 13.36 pg/mL; *P* = 0.003).

### Differential association of plasma MCP-1 levels with markers of inflammation and airway remodeling

Next, we asked if the plasma MCP-1 levels were associated differentially with various inflammatory and airway remodeling biomarkers in diabetic/non-diabetic individuals with and without asthma. To this effect, our data show (Table 2) that in diabetic patients with asthma, MCP-1 levels correlated with IFN-α2, IL-1RA, IL-10, Fractalkine/CX3CL-1, and VEGF whereas, in diabetic patients without asthma, MCP-1 levels correlated with IL-3, IL-6, IL-9, MIP-1α/CCL-3, MIP-1β/CCL-4, GRO-α/CXCL-1, and BMI. In non-diabetic individuals with asthma, MCP-1 levels correlated with IL-1β, IL-1RA, MDC/CCL-22, IP-10/CXCL-10, GM-CSF, FGF-2, PDGF-AA, PDGF-BB, and HbA1c. While, in healthy controls, plasma MCP-1 correlated with IL-1α, IL-1β, IL-2, IL-3, IL-9, BMI, FPG, HbA1c, and TG levels. Altogether, MCP-1 associated differentially with the tested inflammatory and pulmonary remodeling biomarkers and was found to correlate positively with: (i) IFN-α2, IL-10, fractalkine, and VEGF in T2D patients with asthma; (ii) IL-6 and GRO-α in T2D patients without asthma; (iii) MDC, IP-10, GM-CSF, FGF-2, and PDGF-AA/BB in patients with asthma only; and (iv) FPG and TG in non-diabetic non-asthmatic controls. Besides, MCP-1 correlated positively with IL-1RA only in asthma patients, irrespective of their diabetes status.

**Table 2:** Correlation of plasma MCP-1 with immune and clinical biomarkers

Marker	Diabetic				Non-diabetic			
	Asthmatic		Non-asthmatic		Asthmatic		Non-asthmatic	
	r	*P*	r	*P*	r	*P*	r	*P*
IFN-α2	0.62	0.05*	−0.31	0.28	0.38	0.07	−0.31	0.12
IL-1α	−0.37	0.46	0.28	0.64	0.18	0.53	−0.57	0.01*
IL-1β	0.16	0.79	0.53	0.63	0.77	0.002**	−0.62	0.01*
IL-1RA	0.65	0.03*	−0.40	0.17	0.42	0.05*	−0.25	0.21
IL-2	−0.40	0.43	−0.76	0.07	0.51	0.07	−0.49	0.03*
IL-3	0.16	0.74	−0.70	0.05*	−0.17	0.57	−0.78	0.0002***
IL-6	0.24	0.56	0.64	0.04*	0.39	0.11	−0.11	0.62
IL-9	0.16	0.79	−0.85	0.02*	−0.30	0.26	−0.76	0.0004***
IL-10	0.70	0.02*	−0.34	0.26	0.27	0.26	0.18	0.38
IL-12p70	0.22	0.52	−0.48	0.09	0.27	0.23	−0.26	0.20
MDC/CCL-22	−0.01	0.97	0.01	0.96	0.44	0.03*	0.37	0.07
MIP-1α/CCL-3	0.07	0.87	−0.62	0.02*	−0.01	0.96	0.20	0.35
MIP-1β/CCL-4	0.38	0.27	−0.64	0.02*	0.15	0.48	−0.19	0.40
RANTES/CCL-5	−0.02	0.97	0.05	0.90	0.57	0.06	0.01	0.97
GRO-α/CXCL-1	0.47	0.16	0.54	0.05*	0.22	0.30	0.10	0.62
IP-10/CXCL-10	0.57	0.08	0.16	0.61	0.72	<0.0001***	0.18	0.37
Fractalkine	0.70	0.02*	−0.42	0.14	0.28	0.18	−0.30	0.14
G-CSF	0.38	0.27	−0.49	0.08	0.34	0.11	−0.07	0.71
GM-CSF	0.49	0.15	−0.37	0.20	0.54	0.007**	−0.27	0.17
FGF-2	0.40	0.24	−0.12	0.68	0.53	0.01*	0.10	0.62
PDGF-AA	−0.39	0.51	0.11	0.74	0.68	0.04*	−0.39	0.20
PDGF-BB	0.11	0.89	−0.01	0.97	0.91	0.03*	−0.48	0.12
VEGF	0.71	0.02*	−0.005	0.99	0.26	0.21	0.09	0.65
FLT-3 L	−0.30	0.55	−0.61	0.07	0.01	0.99	−0.16	0.50
IgE	0.61	0.06	−0.39	0.38	0.25	0.26	0.19	0.57
BMI (kg/m^2^)	−0.19	0.59	−0.54	0.05*	0.18	0.39	0.40	0.04*
FPG (mmol/L)	−0.11	0.74	0.20	0.51	0.40	0.06	0.46	0.02*
HbA1c (%)	0.03	0.93	−0.24	0.42	0.47	0.02*	0.40	0.05*
TG (mmol/L)	0.25	0.47	−0.12	0.68	0.21	0.31	0.48	0.01*

*IFN* Interferon, *IL* interleukin, *CCL* CC chemokine ligand, *CXCL* CXC chemokine ligand, *MDC* macrophage derived chemokine, *MIP* macrophage inflammatory protein, *RANTES* regulated on activation, normal T-cell expressed and secreted, *GRO* growth regulated oncogene, *IP* IFN-γ-inducible protein, *G-CSF* granulocyte colony-stimulating factor, *GM-CSF* granulocyte-macrophage colony-stimulating factor, *FGF* fibroblast growth factor, *PDGF* platelet derived growth factor, *VEGF* vascular endothelial growth factor, *FLT-3 L* FMS-like tyrosine kinase 3 ligand, *IgE* immunoglobulin E

All statistical values are indicated in boldface

*significant, **highly significant, ***extremely significant

## Discussion

In the present study, significantly elevated plasma MCP-1 levels were observed in T2D patients with asthma (Group I) as compared with other groups i.e., individuals with T2D only (Group II, *P* = 0.03), asthma only (Group III, *P* = 0.05), and non-diabetic non-asthmatic controls (Group IV, *P* = 0.003). Increased MCP-1 levels have been previously reported in T2D individuals [8, 16] as well as in bronchoalveolar lavage fluid from asthmatic patients [17]. Lung is also a target organ for diabetic microangiopathy and the diabetic patients have been reported to be at a higher risk for asthma, chronic obstructive pulmonary disease, pulmonary fibrosis and pneumonia [18]. Mononuclear cells such as lymphocytes and monocytes are involved in systemic and local inflammation in which circulatory monocytes infiltrate into target tissues such as white adipose tissue in case of metabolic inflammation and bronchoalveolar epithelia in asthmatic inflammation. Dandona et al. [13] found the elevated expression of CCR-2 as well as IL-4 and MMP-9 asthma mediators in the mononuclear cell fractions from obese and obese/T2D patients which indicated that these cell populations were responsive to chemotaxis by chemotactic cytokines such as MCP-1/CCL-2 and eotaxin. We speculate that MCP-1 may be a key chemokine involved in monocytic as well as eosinophilic inflammation as both these cell types express cognate CCR-2 receptor. In the present study, MCP-1 levels were found to be significantly higher in diabetic patients with asthma while no significant changes in eotaxin levels could be observed in T2D and/or asthma patients as compared with non-diabetic non-asthmatic controls. It is noteworthy that the presence of asthma in addition to T2D led to a further increase in plasma levels of only the MCP-1 among all biomarkers that we tested in this study which suggests that MCP-1 may be a more sensitive immune marker for disease prognosis in patients inflicted with inflammatory comorbidities, such as T2D and asthma. Hence, the MCP-1 mechanistic link between T2D and asthma may be critical as a risk marker in comorbid inflammatory states. Chemokines play an important role in the pathophysiology of inflammatory diseases including asthma, allergy, atherosclerosis, glomerulonephritis, and obesity/T2D [19]. MCP-1 expression in T2D may be related to the hyperglycemia as high glucose treatment of endothelial cells from diabetic subjects resulted in a 40–70 % increase of MCP-1 and a 10–20 % increase of vascular cell adhesion molecule (VCAM)-1 expression, suggesting a synergistic enhancement due to monocyte-endothelial cell interaction [20]. Similarly, another study showed that high glucose concentrations, advanced glycation end products, glycated albumin, and glycoxidized LDL upregulated MCP-1 expression in human endothelial cells [21]. The higher MCP-1 levels that we found in comorbid (asthmatic diabetic) patients may be due to the reason that both disease conditions involve the underlying etiologic factor of chronic inflammation which may become potentiated by a synergistic response between two morbid states. The previous studies have pointed to an increased risk for asthma in obesity [22] and T2D [18, 23]. We speculate that the obese/T2D individuals with higher systemic and local (broncho-pulmonary epithelial) expression of MCP-1 may be at a high risk and more prone to develop asthma and further studies will be required to verify this line of argument.

In the present study, plasma MCP-1 concentrations were found to be differentially associated with the tested inflammatory, airway remodeling, and allergic biomarkers in diabetic/non-diabetic individuals, with and without asthma. Thus, plasma MCP-1 levels correlated positively with those of: (i) IFN-α2, IL-10, fractalkine, and VEGF in T2D patients with asthma; (ii) IL-6 and GRO-α in T2D patients without asthma; (iii) MDC, IP-10, GM-CSF, FGF-2, and PDGF-AA/BB in asthma only patients; and (iv) FPG and TG in non-diabetic non-asthmatic individuals. This association of MCP-1 with all these biomarkers points to a complex interplay of multiple proteins that leads to the etiopathogenesis of T2D and/or asthma. Notably, the MCP-1 link with both fractalkine and VEGF in T2D patients with asthma reflects its significance as a risk marker in morbid conditions with complex immunologic phenotype of inflammation and angiogenesis. The relationship of MCP-1 with more than one growth factors including GM-CSF, FGF-2, and PDGF as we detected in asthma only patients is in agreement, at least in part, with previous reports showing that FGF-2/PDGF and GM-CSF were detected and important as remodeling and susceptibility biomarkers, respectively, for allergic asthma [24–26]. In contrast to the study reporting a positive correlation between MCP-1 gene expression and BMI in obesity/T2D [27], we did not find a positive association between plasma MCP-1 levels and obesity/T2D clinical markers such as BMI, FPG, HbA1c, and TG in our diabetic cohort. In agreement with our results, Herder et al. reported data from 236 T2D patients, 242 subjects with impaired glucose tolerance, and 244 normoglycemic controls indicating that the MCP-1 levels were not associated with impaired glucose tolerance, T2D or several parameters of obesity [28]. Our data further show that in asthma patients, irrespective of their T2D status, MCP-1 associated positively also with IL-1RA which is an important indicator of the anti-inflammatory competency of these individuals while the IL-1β/IL-1RA balance plays a key role in asthmatic inflammation. The IgE-mediated stimulation of mast cells has been shown to induce IL-1RA expression [29]. Interestingly, in our study, MCP-1 also showed a positive association with IgE levels (*r* = 0.61) in diabetic individuals with asthma; however, it did not approach the level of significance (*P* = 0.06). Overall, our data indicate that plasma MCP-1 levels associate selectively with a wide range of inflammatory cytokines/chemokines, growth/activation factors and could, therefore, be important as a biomarker for asthma in diabetic and non-diabetic subjects while this association of plasma MCP-1 with asthma appears to be driven by the conventional risk factors for inflammation and pulmonary tissue remodeling.

## Conclusion

In summary, our data show that plasma MCP-1 levels were significantly elevated in T2D patients with asthma as compared with those without asthma, and these changes in the systemic MCP-1 related differentially with important biomarkers of inflammation and airway remodeling. Nonetheless, further studies involving larger study cohorts as well as other parameters which might affect inflammation but were not addressed in our study, such as drug therapy will be required to understand the functional significance of MCP-1 in asthma.

## Abbreviations

BMI: Body mass index
CCL: CC chemokine ligand
CXCL: CXC chemokine ligand
ECP: Eosinophil cationic protein
EGF: Epidermal growth factor
ELISA: Enzyme-linked immunosorbent assay
FEV_1_: Forced expiratory volume in 1 sec
FGF: Fibroblast growth factor
FLT-3 L: FMS-like tyrosine kinase 3 ligand
FPG: Fasting plasma glucose
G-CSF: Granulocyte colony-stimulating factor
GM-CSF: Granulocyte-macrophage colony-stimulating factor
GRO: Growth regulated oncogene
HbA1c: Glycated hemoglobin
HDL: High-density lipoprotein
IFN: Interferon
IgE: Immunoglobulin E
IL: Interleukin
IL-1RA: IL-1 receptor agonist
IP: IFN-γ-inducible protein
LDL: Low-density lipoprotein
MCP-1: Macrophage chemoattractant protein-1
MDC: Macrophage derived chemokine
MIP: Macrophage inflammatory protein
OGTT: Oral glucose tolerance test
PDGF: Platelet-derived growth factor
RANTES: Regulated on activation, normal T-cell expressed and secreted
sCD40L: soluble CD40 ligand
T2D: Type-2 diabetes
TG: Triglycerides
TGF: Transforming growth factor
VCAM-1: Vascular cell adhesion molecule-1
VEGF: Vascular endothelial growth factor
