# Erratum to: ‘Differential association of plasma monocyte chemoattractant protein-1 with systemic inflammatory and airway remodeling biomarkers in type-2 diabetic patients with and without asthma’

**DOI:** 10.1186/s40200-016-0270-6

**Published:** 2016-10-12

**Authors:** Sardar Sindhu, Merin Koshy, Areej Abu Al-Roub, Nadeem Akhter, Saad Al Zanki, Shamsha Ali, Sriraman Devarajan, Rasheed Ahmad

**Affiliations:** 1 Immunology & Innovative Cell Therapy Unit, Dasman, Kuwait; 2 Tissue Bank Facility, Dasman Diabetes Institute (DDI), Dasman, Kuwait

Unfortunately, the original version of this article [1] contained an error. The acknowledgements was included incorrectly. The complete acknowledgements can be found below:

We thanks all our participants for their voluntary participation, cooperation, and support. The authors thank the staff of pancreatic islet biology and transplantation unit for help with Luminex assays.

## References

[1] Sindhu S, Koshy M, Al-Roub AA, Akhter N, Al Zanki S, Ali S, Devarajan S, Ahmad R (2016). Differential association of plasma monocyte chemoattractant protein-1 with systemic inflammatory and airway remodeling biomarkers in type-2 diabetic patients with and without asthma. J Diabetes Metab Disord.

